# Isolated massive pleural effusion as a manifestation of chronic graft versus host disease successfully treated with corticosteroid

**DOI:** 10.1007/s00277-024-05643-w

**Published:** 2024-01-29

**Authors:** Yasutaka Masuda, Sho Yamazaki, Akira Honda, Yosuke Masamoto, Mineo Kurokawa

**Affiliations:** 1 Department of Hematology and Oncology, Graduate School of Medicine, The University of Tokyo, 7-3-1 Hongo, Bunkyo-Ku, Tokyo, 113-8655 Japan; 2 Department of Cell Therapy and Transplantation Medicine, The University of Tokyo Hospital, 7-3-1 Hongo, Bunkyo-Ku, Tokyo, 113-8655 Japan

**Keywords:** Allogeneic hematopoietic stem cell transplant, Chronic graft versus host disease, Pleural effusion

## Abstract

Isolated pleural effusion is a rare manifestation of chronic graft versus host disease (cGVHD) after hematopoietic stem cell transplantation (HSCT). We herein report a 58-year-old woman presenting with massive pleural effusion approximately 1 year after allogeneic HSCT, who was successfully treated with corticosteroid. She had discontinued tacrolimus approximately 1 month before she presented with pleural effusion, which was attributed to cGVHD after a thorough exclusion process. This case illustrates a unique manifestation of atypical cGVHD and highlights the need for prompt therapy initiation.

## Introduction

Pulmonary complications of allogeneic hematopoietic stem cell transplantation (allo-HSCT) occur in as many as 60% of stem cell recipients and pose a diagnostic and therapeutic challenge to clinicians [1–5]. Pleural effusion after allogeneic HSCT can be caused by many reasons, including infection, volume overload, serositis-type graft versus host disease (GVHD), and heart failure. Pleural effusion has been associated with significantly worse overall survival and pulmonary-related mortality in a recent cohort [6]. Clinical features of chronic GVHD (cGHVD)-associated pleural effusion have not been extensively studied because of its rarity, but a few studies indicate that it occurs mainly after other cGVHD manifestations and after immunosuppressants taper [6, 7].

We herein report a 58-year-old woman presenting with sudden development of isolated massive pleural effusion approximately 1 year after receiving allo-HSCT, who was successfully treated with corticosteroid.

## Case report

A 58-year-old woman who had received cord blood transplantation (CBT) from a 7/8 HLA-matched donor for myelodysplastic syndromes 314 days before presented at an outpatient clinic with a complaint of generalized edema and worsening dyspnea for the past week. Prior to the transplantation, she had received no chemotherapies. She was preconditioned with a myeloablative regimen (fludarabine 30 mg/m^2^ × 5 days, melphalan 140 mg/m^2^ × 1 day, and total body irradiation 2 Gy × 2 times) and was given short-term methotrexate and continuous tacrolimus as GVHD prophylaxis. Neutrophil engraftment was achieved on day 30. On day 34, 1 mg/kg prednisolone treatment was started for noncardiogenic pulmonary edema suspected of engraftment syndrome, which improved within a week. She had no acute GVHD symptoms. After day 100, mild skin rash considered cGVHD appeared (skin score 1 according to NIH criteria [8]). No other cGVHD symptoms occurred and skin rash improved; therefore, on day 274, tacrolimus was discontinued. Two months prior to admission, she noticed edema of the lower limbs, and oral furosemide was prescribed by her family physician. The edema had exacerbated steadily and was not responsive to oral furosemide intake, which was stopped soon after by our outpatient attending physician. Subsequently, she experienced mild anorexia but did not experience nausea, vomiting, or diarrhea.

**Fig. 1:**
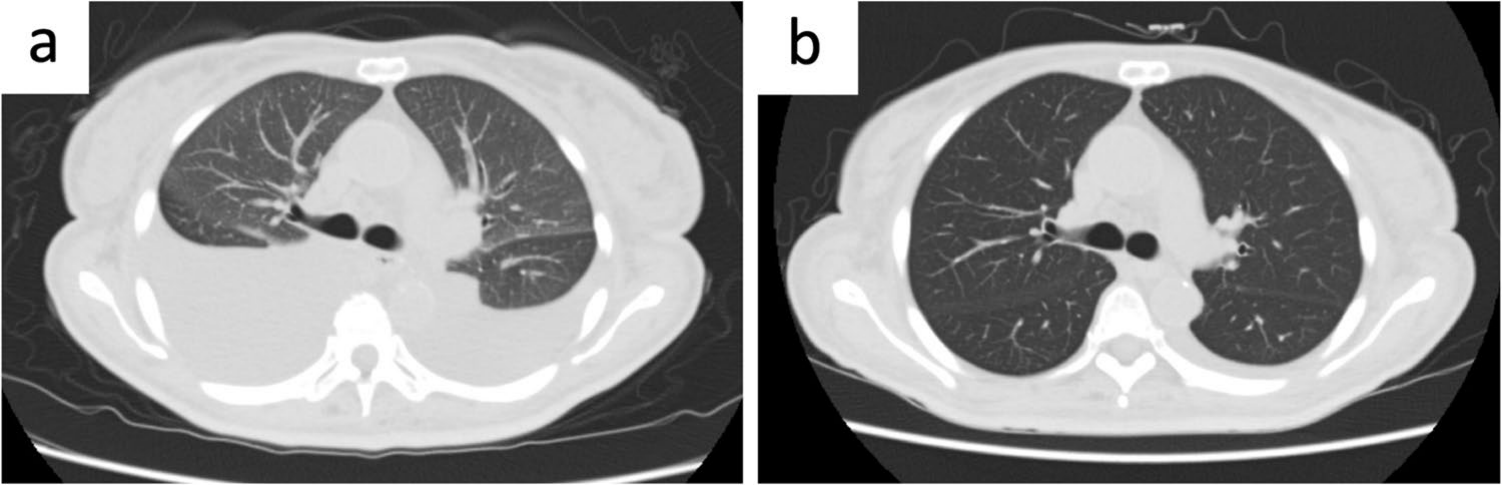
CT at admission (**a**) and on 12 days after admission (**b**)

**Fig. 2:**
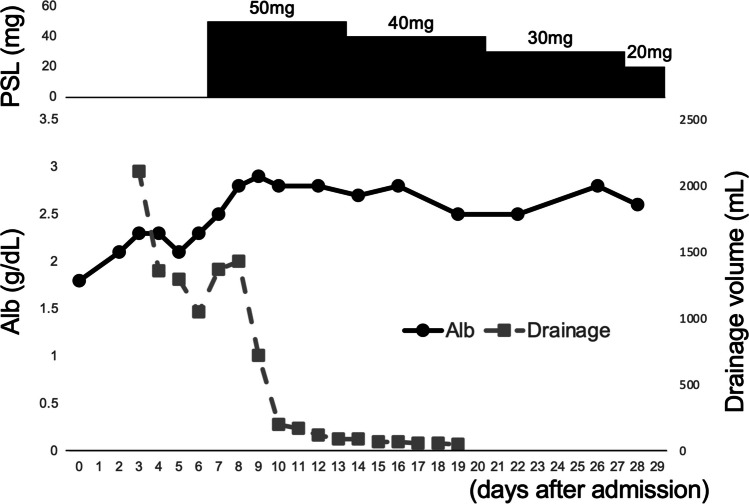
Clinical time course of the present case. Alb, albumin; PSL, predonisolone

On admission, generalized pitting edema, especially of lower limbs, and orthopnea were observed. Her medications on admission were levothyroxine, vonoprazan fumarate, ursodeoxycholic acid, acyclovir, and linagliptin. Her body weight had increased from 60 kg to 69 kg for the last 3 months. Computed tomography (CT) revealed generalized subcutaneous edema and massive bilateral pleural effusion (Fig. 1a). Neither abdominal ascite nor pericardial effusion was apparent. Bilateral lung fields did not show inflammatory changes on CT, and serum virus screening was negative (cytomegalovirus antigenemia, respiratory syncytial virus antibody, herpes simplex virus antibody, varicella zoster virus antibody, coronavirus disease 2019 PCR, adenovirus antibody, parvovirus B19 antibody). Transthoracic echocardiography showed normal cardiac contractility with an ejection fraction of 64% but slight right heart overload with a calculated tricuspid regurgitation peak gradient of 35 mmHg and right ventricular systolic pressure of 44 mmHg. The levels of brain natriuretic peptide (BNP) were elevated (52.8 pg/mL) but were stable compared to previous results (BNP was 177.9 pg/mL 3 months prior to current admission). She had no cardiovascular history. These findings directed us to investigate noncardiac causes of pleural effusion. Albumin, which was low on admission (1.8 g/dL) and was considered a contributing factor to edema and effusion, was supplemented intravenously. Although vitamin B1 level was low (19 ng/mL, reference range 24 to 66) and we empirically administered a total of 300 mg thiamine for the first few days, characteristic findings of wet beriberi, namely, high ejection fraction and elevated lactate (0.7 mmol/L on day 3 after admission), were not observed, making beriberi unlikely. Thoracentesis was performed, revealing transudative pleural effusion according to Light’s criteria, with total protein 2.4 g/dL and 5.5 g/dL (reference range, 6.6 to 8.1), and lactate dehydrogenase 94 U/L and 253 U/L (reference range, 124 to 222) in pleural effusion and serum, respectively. Flow cytometry and cell block preparation analysis of effusion identified no malignant cells but abundant lymphocytes, approximately 66% of which comprised CD3+ T cells with a predominance of CD8+ cells over CD4+ cells (CD4/8 ratio: 0.61). Neither bacterial culture nor mycobacterium PCR was positive. Two days after admission, she was transferred to the intensive care unit, where three chest drains were inserted, and non-invasive positive pressure ventilation (NPPV) was started since oxygen demand increased. Although the albumin level increased with supplementation, we failed to lower the NPPV setting, consistent with persistently low pulmonary transparency on chest radiographs and stably large (1000 to 1500 mL per day) drainage volume from chest tubes. These findings and time course prompted us to consider cGVHD as the etiology of pleural effusion. No other cGVHD symptoms were apparent. Normal random urine protein/creatinine ratio (0.3 g/gCr), normal lactate dehydrogenase, elevated haptoglobin, and absence of de novo anemia and thrombocytopenia made thrombotic microangiopathy-associated (TMA) pleural effusion unlikely (Table 1). Prednisolone (1 mg/kg, 50 mg/body) administration was thus initiated on the seventh hospital day (321 days after CBT). After introducing steroids, drainage volume from chest tubes rapidly decreased to approximately 100 mL within a week, corresponding to the disappearance of pleural effusion on CT (Figs. 1b and 2). The prednisolone dose was tapered gradually to 20 mg 24 days after steroid initiation. All chest tubes were removed, and her edema reduced. For lingering mild edema, she was prescribed with azosemide and spilonolactone at discharge. She was discharged on day 343 after CBT.

**Table 1:** Laboratory examinations on admission

Blood chemistry	Value	Reference range	Complete blood count	Value	Reference range
Total protein	5.5	6.6–8.1 g/dL	White cell count	8.3	3.3–8.6 × 10^3^/µL
Albumin	1.8	4.1–5.1 g/dL	Red cell count	305	386–492 × 10^5^/µL
Prealbumin	5.3	22–34 mg/dL	Hemoglobin	9.7	11.6–14.8 g/dL
C-reactive protein	0.49	0–0.3 mg/dL	Hematocrit	30.2	35.1–44.4%
Lactate dehydrogenase	253	124–222 U/L	Platelets	17.8	15.8–34.8 × 10^5^/µL
Aspartate aminotransferase	19	13–30 U/L			
Alanine aminotransferase	16	7–23 U/L	Coagulation		
γoagulation otransferases	212	9–32 U/L	PT%	88.5	86–124.1%
Alkaline phosphatase	147	38–113 U/L	APTT	38.3	24–34 s
Total bilirubin	0.5	0.4–1.5 mg/dL	Fibrinogen	276	168–355 mg/dL
Urea nitrogen	9.8	8–20 mg/dL	D dimer	3.8	0–1 µg/mL
Creatinine	0.91	0.46–0.79 mg/dL			
Sodium	138	138–145 mmol/L	Trace elements		
Potassium	4.1	3.6–4.8 mmol/L	Iron	12	40–188 µg/dL
Chloride	108	101–108 mmol/L	Copper	55	70–132 µg/dL
Magnesium	1.5	1.8–2.4 mg/dL	Ceruloplasmin	10.7	21–37 mg/dL
Vitamin B1	19	24–66 ng/mL	Zinc	29	80–130 µg/dL
sIL-2R	999	127–582 U/mL			

*APTT* activated partial thrombin time, *sIL-2R* soluble interleukin 2 receptor, *PT* prothrombin time

## Discussion

Polyserositis is a rare and atypical manifestation of cGVHD, occurring in approximately 1% of patients undergoing allo-HSCT [9]. It is a diagnosis of exclusion, and is particularly challenging when other cGVHD manifestations are lacking.

In our present case, pleural effusion was primarily ascribed to cGVHD because other etiologies were unlikely. Normal cardiac contractibility on transthoracic echocardiography, negative virus screening results, and the negative culture of peripheral blood as well as pleural effusion for infectious causes made congestive heart failure and infection unlikely for the primary cause of pleural effusion. Malignancy was also ruled out on cell block preparation and flow cytometry of effusion. Pleural effusion is one of the known manifestations of transplant-associated TMA [10]; however, laboratory findings indicated minimal involvement of TMA. These exclusion processes led us to ascribe pleural effusion to cGVHD. The resistance of pulmonary effusion to albumin supplementation and rapid responsiveness to steroid administration supported our assumption of cGVHD-associated serositis, also making hypoalbumin-induced anasarca unlikely. Indeed, the etiology of hypoalubumin is of another interest (1.8 g/dL on admission). This might be due to insufficient protein intake or due to renal or gastrointestinal loss. A nutrient deficiency was corroborated by a low level of rapid turnover protein prealbumin, vitamin B1, iron, and other trace elements (zinc, copper, and ceruloplasmin; Table 1). Although she did not experience nausea, vomiting, or diarrhea, gastrointestinal GVHD might have had occurred concurrently. Urinalysis revealed proteinuria; however, urine protein creatinine ratio never exceeded 2.5 g/gCr, and abnormal urinary casts were never detected; thus, renal involvement was deemed questionable. Interestingly, a significant decrease in serum albumin was observed in patients with cGVHD-associated serositis [7]. Thus, low albumin in our present case might possibly be due to severe systemic illness as a negative acute phase reactant.

Due to its rarity, pleural effusion as a chronic complication of allo-HSCT has not been extensively studied. In a retrospective single-center analysis, Leonard et al. reported that 20 (1.8%) patients developed cGVHD-associated serositis out of 946 patients undergoing allo-HSCT [7]. They found that cGVHD-associated serositis occurs mainly following immunosuppressant taper (15/20 cases), and most patients had a prior diagnosis of cGVHD (17/20 cases) [7]. Pleural effusion occurred in 15 patients, with only one patient developed without concomitant pericarditis. A similar incidence was reported by another single-center cohort, whereby eight patients (1.3%) developed cGVHD-associated serositis with a median of approximately 400 days after allo-HSCT [6]. Out of these eight patients, four had concomitant pericardial effusions, and two had ascites. Of note, all these patients had continued to take immunosuppressants at the onset of pleural effusion. These findings suggest that de novo pleural effusion without prior cGVHD manifestation and/or without prior immunosuppressive medications is extremely rare, and the majority cases of pleural effusion occur with other serositis manifestations. In our present case, tacrolimus was maintained at a low dose for mild skin rash and was discontinued approximately 1 month before she started to express dyspnea; therefore, cessation of the immunosuppressant could be responsible for the occurrence of these symptoms. Pleural effusion occurred without apparent other serositis or cGVHD symptoms, making this case a unique manifestation.

The pathophysiology of cGVHD-associated pleural effusion is even more obscure. Flow cytometry of the effusion sample of our case identified an abundance of CD8 + -skewed T lymphocytes, consistent with a previous report [11]. In that study, thickening of the pleural and infiltration of CD3 + T cells with fewer CD25 + regulatory T cells was identified on pleural biopsy [11]. Although exudate rather than transudate feature of pleural effusion generally indicates pleural inflammation [12], GVHD-associated serositis could be transudate as well as exudate in previous reports [13, 14]. Characterizing graft versus host immunological interplay at pleura and in effusion is of future studies.

## Conclusion

In conclusion, this case illustrates isolated pleural effusion associated with cGVHD, which was steroid responsive. Our case also highlights that cGVHD could present as an isolated pleural effusion without other apparent cGVHD manifestations. The diagnosis requires thorough exclusion of other etiologies such as congestive heart failure, infection, and TMA, among others. In spite of the difficulty in the diagnosis, pleural effusion associated with cGVHD could be massive and life threatening, and therefore necessitates swift treatment initiation. Hematologists in charge of post-HSCT follow-up are encouraged to be particularly vigilant to the patient’s status after immunosuppressant discontinuation, and should be suspicious of cGVHD even when the pleural effusion occurs without other apparent serositis manifestations.

## Data Availability

Data used in this manuscript is not publicly available in order to protect patient confidentiality. Reasonable request should be directed to the corresponding author.
